# Age-related changes in lysosomal abundance in mouse hearts assessed by Lysotracker fluorescence imaging and autophagy gene expression analysis

**DOI:** 10.1007/s10554-026-03719-4

**Published:** 2026-05-19

**Authors:** Jawaher Albulushi, Hannah Coghlan, Mohesh Moothanchery, Aiswarya Dev, Emily Akerman, Jasmine Heenan, Nordine Helassa, Oluwatobi Adegbite, Parveen Sharma, Fenil Patel, Libby Harrison, Mahon L. Maguire, Gary R. Mirams, Pawel Swietach, Harish Poptani, Rebecca AB Burton

**Affiliations:** 1 Institute of Systems, Molecular and Integrative Biology, Department of Pharmacology and Therapeutics, University of Liverpool, Liverpool, UK; 2 Centre for Preclinical Imaging, Liverpool Shared Research Facilities, University of Liverpool, Liverpool, UK; 3 SAINBIOSE U1059, INSERM, Université Jean Monnet, Mines Saint-Etienne, Saint-Etienne, France; 4 Institute of Systems, Molecular and Integrative Biology, Department of Biochemistry, University of Liverpool, Liverpool, UK; 5 Institute of Life Course and Medical Sciences, Department of Cardiovascular and Metabolic Medicine, University of Liverpool, Liverpool, UK; 6 Liverpool Centre for Cardiovascular Science, University of Liverpool, Liverpool John Moores University and Liverpool Heart and Chest Hospital, Liverpool, UK; 7 Centre for Mathematical Medicine & Biology, School of Mathematical Sciences, University of Nottingham, Nottingham, UK; 8 Department of Physiology, Anatomy and Genetics, University of Oxford, Oxford, UK

**Keywords:** Calcium, IVIS, Lysotracker, Ageing, Heart

## Abstract

**Purpose:**

Lysosomal function is essential for cardiac proteostasis and cellular health, yet its regulation during ageing remains poorly defined. We aimed to determine whether whole-organ, fluorescence imaging using an In Vivo Imaging System (IVIS) provides a novel, rapid and scalable approach for quantifying lysosomal abundance in intact *ex vivo* hearts prior to deeper molecular analysis.

**Methods:**

*Ex vivo* hearts from young (2–4 months) and aged (18 months) mice were labelled with Lysotracker™ Red and imaged using IVIS, to quantify whole-heart acidic-vesicle-associated fluorescence signals. Expression of lysosomal and autophagy-related genes (*Lamp2*, *Atp6v1a*, *Sqstm1*, *Cd63*, *Atg12*, *Nfe2l2*, *M6pr*) was assessed by RT-qPCR.

**Results:**

Whole-heart Lysotracker fluorescence did not differ significantly between age groups, indicating preservation of overall acidic-vesicle pool. Expression of *Atp6v1a* and *Lamp2* was unchanged, suggesting maintained acidification capacity and lysosomal structure, whereas minor, upregulation of *Sqstm1* might indicate increased autophagic demand and altered vesicle trafficking, which warrants further investigation. No statistically significant changes in *M6pr*, *Atg12*, or *Nfe2l2* were detected, suggesting transcriptional stability in enzyme trafficking, core autophagy, and oxidative stress pathways. Regionally, atria showed higher Lysotracker signal than ventricles, consistent with known enrichment of acidic vesicular stores in atrial physiology.

**Conclusion:**

IVIS-based Lysotracker imaging provides a rapid whole-organ approach for assessing acidic vesicle distribution in intact hearts, enabling scalable screening of lysosome-associated physiology. While limited by depth-dependent optical attenuation and lack of organelle specificity, this approach complements molecular analysis and supports integrated investigation of lysosomal and autophagy pathways during cardiac ageing.

**Graphical Abstract:**

**Figure:**
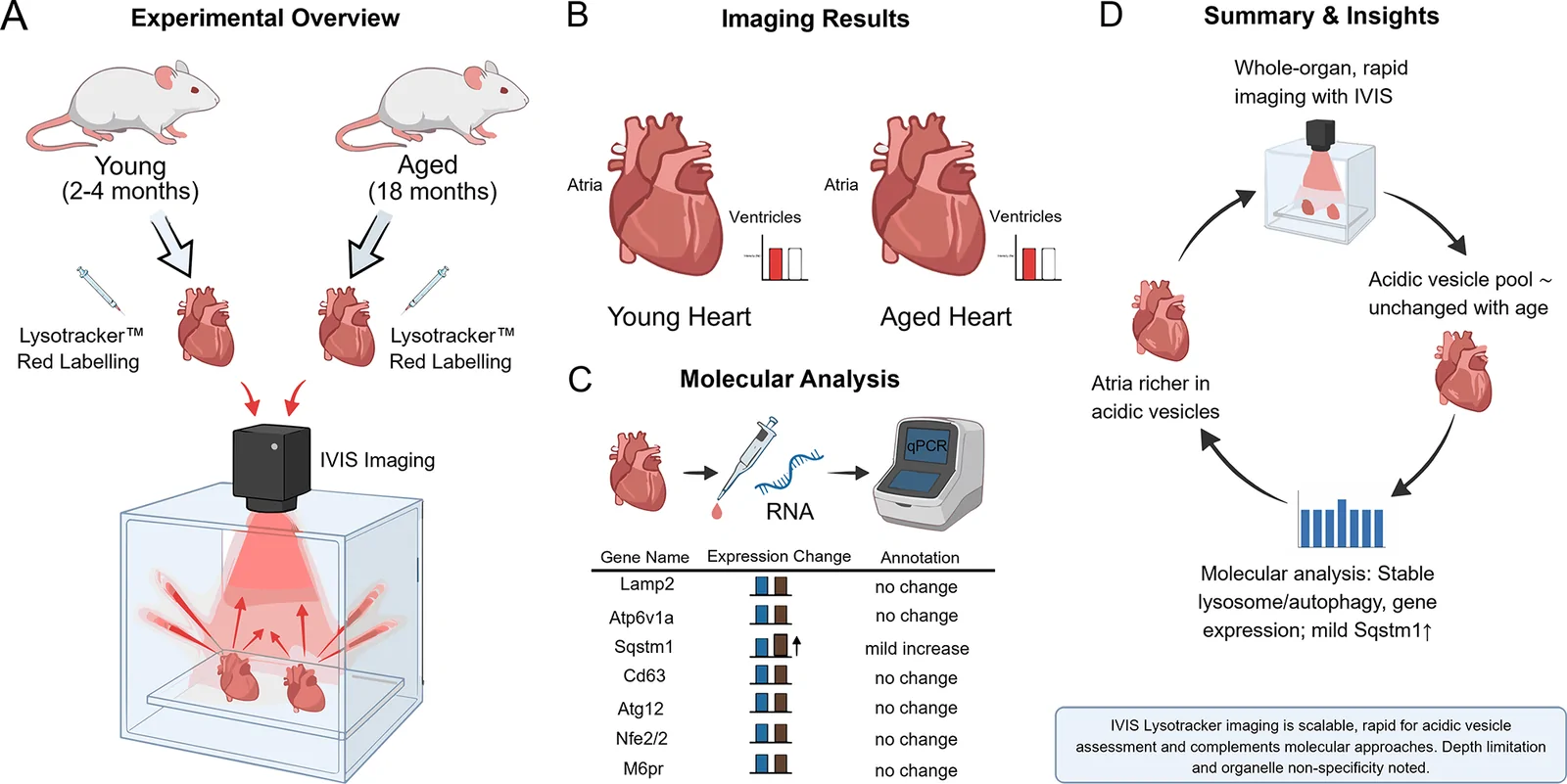

**Supplementary Information:**

The online version contains supplementary material available at https://doi.org/10.1007/s10554-026-03719-4.

## Introduction

Ageing is an inevitable biological process and the most significant non-modifiable risk factor for cardiovascular disease (CVD) [1]. As the population ages, the burden of CVD is projected to rise markedly. Between 2025 and 2050, cardiovascular deaths are expected to increase from 20.5 million to 35.6 million, a 73.4% rise, driven by population growth and global ageing [2]. Although age-standardized mortality rates are anticipated to decline by approximately 30.5%, reflecting improvements in medical care following diagnosis, the absolute number of cases and deaths will continue to grow, underscoring the need for targeted healthcare strategies in ageing populations [2].

In humans, the ageing heart undergoes both structural and functional changes, including thickening of the left ventricle (LV), diastolic dysfunction, fibrosis, and reduced cardiac output under stress [1, 3]. Even without comorbidities such as hypertension or diabetes, these intrinsic alterations elevate mortality risk by diminishing cardiac efficiency and adaptability [3]. At the cellular level, ageing is characterized by elevated oxidative stress, mitochondrial dysfunction, reduced metabolic flexibility, and increased cardiomyocyte senescence [1, 4]. These changes compromise energy homeostasis and calcium (Ca^2+^) handling, contributing to progressive cardiac impairment. Increasing attention has focused on the failure of cellular quality control systems, particularly autophagy and lysosomal degradation, which are essential for clearing damaged proteins and organelles [4, 5].

Historically regarded as passive sites of cellular waste disposal, lysosomes were first identified in 1955 by Christian de Duve through subcellular fractionation [6], a discovery that laid the foundation for modern cell biology. They are now recognized as dynamic organelles with critical roles in metabolic regulation, intracellular signalling, and inter-organelle communication [7]. This evolving understanding has positioned lysosomes as central players in the pathophysiology of ageing and chronic diseases [7].

Within cardiomyocytes, lysosomes have emerged as key regulators of Ca^2+^ homeostasis, energy metabolism, and cellular stress responses [8, 9]. Further studies show that lysosomal Ca^2+^ stores are essential for modulating how heart cells respond to stress signals like β-adrenergic stimulation [8]. Two-pore channel (TPC) family, TPC-type2 (TPC2) appears to be the dominant channel involved in generating these nicotinic acid adenine dinucleotide phosphate (NAADP)-triggered Ca^2+^ signals [8]. The use of pharmacological agents such as bafilomycin A1 and glycyl-L-phenylalanine 2-naphthylamide (GPN) has provided functional evidence that NAADP-evoked Ca^2+^ signals originate specifically from lysosomes [10].

Additionally, during ischemia–reperfusion injury (a type of damage that occurs when blood returns to the heart after a period of oxygen deprivation), NAADP-mediated signalling triggers Ca^2+^ release from lysosomes. This lysosomal Ca^2+^ release promotes pathological Ca^2+^ oscillations that drive mitochondrial Ca^2+^ overload and ultimately cardiomyocyte death [11]. Pharmacological inhibition of NAADP signalling at the onset of reperfusion significantly reduced infarct size and improved cardiac outcomes in experimental models, implicating lysosomal Ca^2+^ release as an early initiator of reperfusion-induced injury [11].

Beyond their role in Ca^2+^ signalling, lysosomes are essential regulators of cellular energy balance and protein quality control. They closely interact with mitochondria and influence key pathways such as autophagy and mTOR signalling, both of which are frequently disrupted during cardiac ageing and disease [1, 12]. Impaired lysosomal acidification or blocked autophagic flux can trigger pathological conditions, including myocardial infarction and cardiac hypertrophy [1, 12]. In the ageing myocardium, particularly in long-lived, non-dividing cardiomyocytes, lysosomal degradative capacity declines, leading to the buildup of oxidized proteins, dysfunctional organelles, and lipofuscin. This overload compromises mitochondrial recycling and reduces the heart’s contractile function and stress resilience over time [4, 5, 12].

Atrial fibrillation (AF) is one of the most common arrhythmias, and its prevalence increases markedly with ageing. Endolysosomal dysregulation as well as lysosomal spatial reorganisation [9, 13] has been observed in experimental models of AF, suggesting a potential mechanistic link between ageing-related cellular changes and arrhythmogenesis [14]. This suggests that lysosomes and related acidic compartments may play a role in the structural and metabolic remodelling seen in AF [14]. These findings support the idea that lysosomes serve as both sensors and effectors of cardiac stress, that may drive disease progression [7].

Recent research has substantially advanced our understanding of lysosomal dysfunction in CVDs, particularly in acute injury setting such as myocardial ischemia-reperfusion injury (MI/RI), as well as in conditions including diabetic cardiomyopathy and lysosomal storage disorders [15–19]. However, opportunities remain to deepen our knowledge of lysosomal involvement in ageing-associated chronic cardiac remodelling. While most studies have focused on the acute stress response, emerging evidence highlights the importance of understanding lysosomal regulation in the ageing heart and its contribution to progressive myocardial decline [15, 20]. Promising strategies such as the transcription factor EB (TFEB) activation (a master regulator of lysosomal biogenesis and autophagy), autophagy modulation, and gene therapy have shown considerable potential for restoring lysosomal function [15–20]. Expanding these approaches to age-related cardiomyopathies represents an exciting avenue for future research, with the possibility of developing targeted, lysosome-based therapeutic interventions.

Among emerging therapeutic strategies, targeting lysosomal Ca^2+^ signalling has gained particular attention. Modulation of two-pore channels – type 2 (TPC2) has demonstrated anti-arrhythmic effects in models of catecholaminergic stress by suppressing aberrant Ca^2+^ release events [8]. While these findings highlight the potential of lysosome-based interventions for cardiac electrophysiology, their physiological relevance during age-related structural and metabolic remodelling remains to be fully understood. In parallel, combinatorial approaches integrating TFEB activators, autophagy modulators, and gene therapies are showing increasing promise; however, their application to the ageing myocardium remains in the early experimental stages [20].

Imaging technologies have advanced lysosomal research, with fluorescence microscopy providing high spatial resolution and molecular specificity. However, these approaches are often limited to cellular or subcellular imaging scales and may require invasive sample preparation, restricting their ability to capture whole-organ physiology. Non-invasive imaging modalities such as magnetic resonance imaging (MRI), positron emission tomography (PET), and computed tomography (CT) provide valuable whole-organ structural and functional information, with MRI offering excellent soft-tissue contrast, PET high metabolic sensitivity, and CT detailed anatomical resolution. However, these techniques lack the molecular specificity required to directly assess lysosomal compartments [21–23]. Emerging approaches such as chemical exchange saturation transfer (CEST) MRI show potential for probing lysosomal pH but remain limited by sensitivity, motion artefacts, and the need for specialised contrast agents [23]. In contrast, in vivo imaging systems (IVIS) enable rapid, non-destructive optical imaging of intact tissues and organs using fluorescent or bioluminescent probes. IVIS is widely used in small animal research for longitudinal and whole-body imaging, offering high sensitivity and throughput. Despite these advantages, its application to cardiac lysosomal biology remains underexplored. Optical imaging with IVIS is inherently limited by light scattering and absorption [22], resulting in depth-dependent signal attenuation and reduced spatial resolution in dense tissues such as the heart.

Importantly, IVIS provides a unique opportunity to bridge the gap between cellular-level assays and whole-organ physiology. By enabling rapid assessment of probe distribution across intact organs, it allows semi-quantitative evaluation of biological processes such as acidic vesicle abundance, metabolic activity, and oxidative stress in a spatially integrated manner. In cardiac research, this is particularly valuable given the structural and functional heterogeneity of the myocardium. When combined with targeted fluorescent probes such as Lysotracker, IVIS offers a scalable platform for screening lysosome-associated processes prior to more detailed molecular or high-resolution imaging analyses.

In this study, we hypothesised that ageing would alter the abundance of acidic organelles and their acidification machinery in cardiac tissue. To test this, we used IVIS fluorescence imaging of Lysotracker to quantify whole-heart Lysotracker uptake as a proxy for the pool of acidic vesicles [24], and real-time polymerase chain reaction (RT-qPCR) to assess key lysosomal genes, including *Lamp2* (lysosomal structure), *Cd63* (late endosome/multivesicular body (MVB) marker), *M6pr* (endosomal trafficking), *Atg1*2 (autophagy initiation), *Sqstm1* (autophagy adaptor), *Nfe2l2* (oxidative stress regulator), and *Atp6v1a* (a v-ATPase subunit essential for lysosomal acidification) [25]). This approach positions IVIS as a rapid, non-destructive, whole-organ imaging platform that complements traditional microscopy and molecular assays for studying lysosomal biology.

## Results

To assess whether lysosomal abundance changes with age in the heart, we compared Lysotracker fluorescence signal in *ex vivo* hearts from young and old mice (3 animal batches, *n* = 7 per age group) using IVIS imaging. Quantitative analysis of total cardiac fluorescence revealed no significant difference between age groups (*p* > 0.05). Given the surface-weighted nature of optical imaging, these measurements could primarily reflect lysosomal signal from superficial myocardial layers and should be interpreted as semi-quantitative. Because optical excitation and emission are exponentially attenuated in biological tissue, planar fluorescence images are inherently surface-weighted. This effect is more pronounced at shorter visible wavelengths (< 600 nm), where absorption and scattering are greater than in the near-infrared (NIR) range [26]. Thus, while our data suggest comparable epicardial lysosomal activity between age groups, we cannot exclude age-related changes in deeper myocardial regions that may be underestimated due to depth-dependent attenuation. Additionally, age-associated alterations in myocardial structure or optical properties could influence fluorescence detection independently of lysosomal biology. Together, these findings indicate that global myocardial Lysotracker signal appears preserved with ageing under our imaging conditions, though complementary *ex vivo* or histological analyses will be required to fully assess potential transmural or cell-type–specific differences. To complement these findings, we measured the expression of the lysosomal membrane protein gene *Lamp2* by RT-qPCR in whole-heart RNA extracts. Consistent with the imaging results, *Lamp2* transcript levels did not differ significantly between young and aged hearts. Together, these data suggest that gross lysosomal abundance is maintained in the ageing heart, although subtle or region-specific alterations may not be captured by surface-weighted imaging or bulk transcript analysis.

### IVIS Imaging

**Fig. 1:**
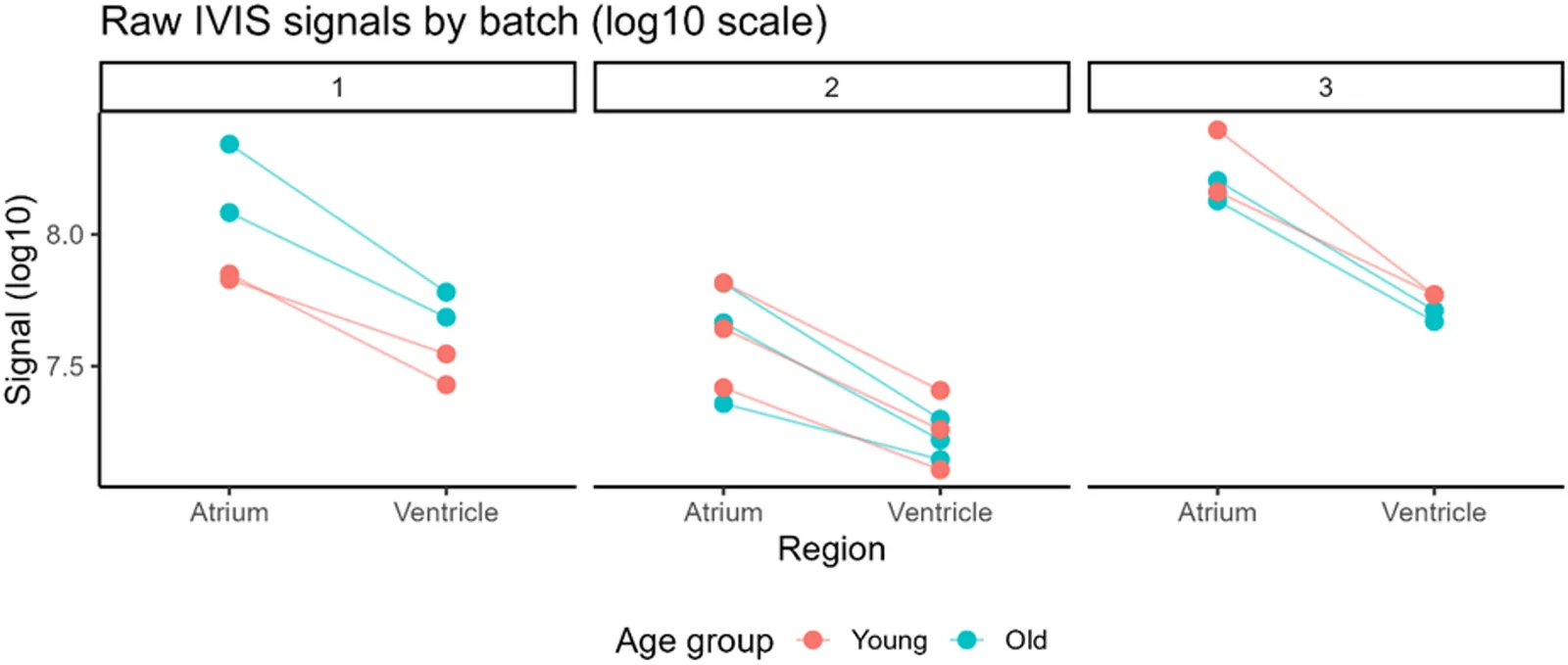
Raw Lysotracker fluorescence IVIS signals by batch (log10 scale). Raw IVIS signals (log10 scale) for atrium and ventricle in young and old mice, shown separately for each imaging batch. Lines connect atrium and ventricle values from the same mouse. In some batches, old mice appeared to have slightly higher or lower signals than young mice, but these differences were inconsistent in direction across batches. Old animals *n* = 7, young animals *n* = 7

Animals were purchased in three batches (see Methods). Visual inspection of the raw IVIS data (Fig. 1) showed that atrial signals were consistently higher than ventricular signals in both age groups. In batch 2, old mice appeared to have lower signals than young mice. Overall signal intensity also varied between batches.

**Fig. 2:**
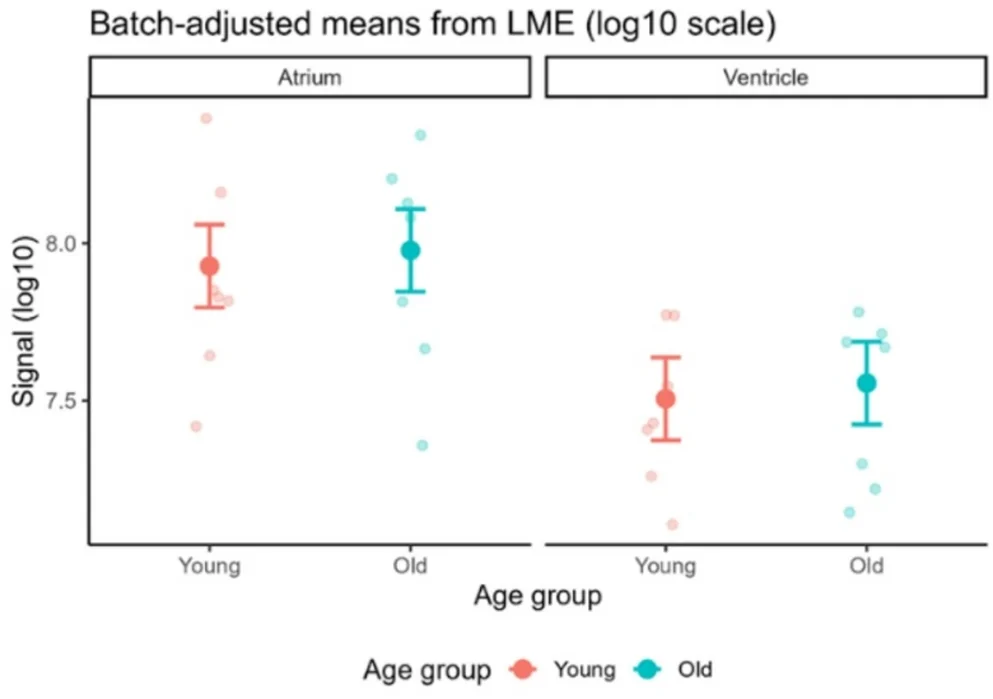
Batch-adjusted means (log10 scale) from the linear mixed-effects model for atrium and ventricle in young and old mice. Points represent estimated marginal means (± 95% CI) after adjusting for batch effects; faint points show individual mouse values. Batch-adjusted means indicate higher atrial signals than ventricular signals in both age groups, with overlapping 95% confidence intervals between young and old mice

Given these patterns, and the paired nature of the data (atrium and ventricle from the same mouse), a linear mixed-effects model was used (see Supplementary Table S1 for full estimated marginal means (EMMs), Supplementary Figure S1 for diagnostic plots (i.e. model-checking plots assessing assumptions of the linear mixed-effects model), and Supplementary Table S2 for the full ANOVA results), with Age group, Region, and Batch as fixed effects, and Mouse ID as a random effect. The analysis confirmed a significant effect of Region (F(1,13) = 207.31, *p* < 0.0001) and Batch (F(2,10) = 17.30, *p* = 0.0006), but no significant effect of Age group (F(1,10) = 0.38, *p* = 0.553). Batch-adjusted means (Fig. 2) indicated that, on the log10 scale, atrial signals averaged between 7.93 and 7.98 for young and old mice, whereas ventricular signals averaged between 7.51 and 7.56. Back-transformed geometric means correspond to ~ 8.45–9.48 × 10^7^ photons/sec/cm²/sr for atria and ~ 3.21–3.60 × 10^7^ for ventricles, with overlapping 95% confidence intervals between age groups.

### RT-qPCR

**Fig. 3:**
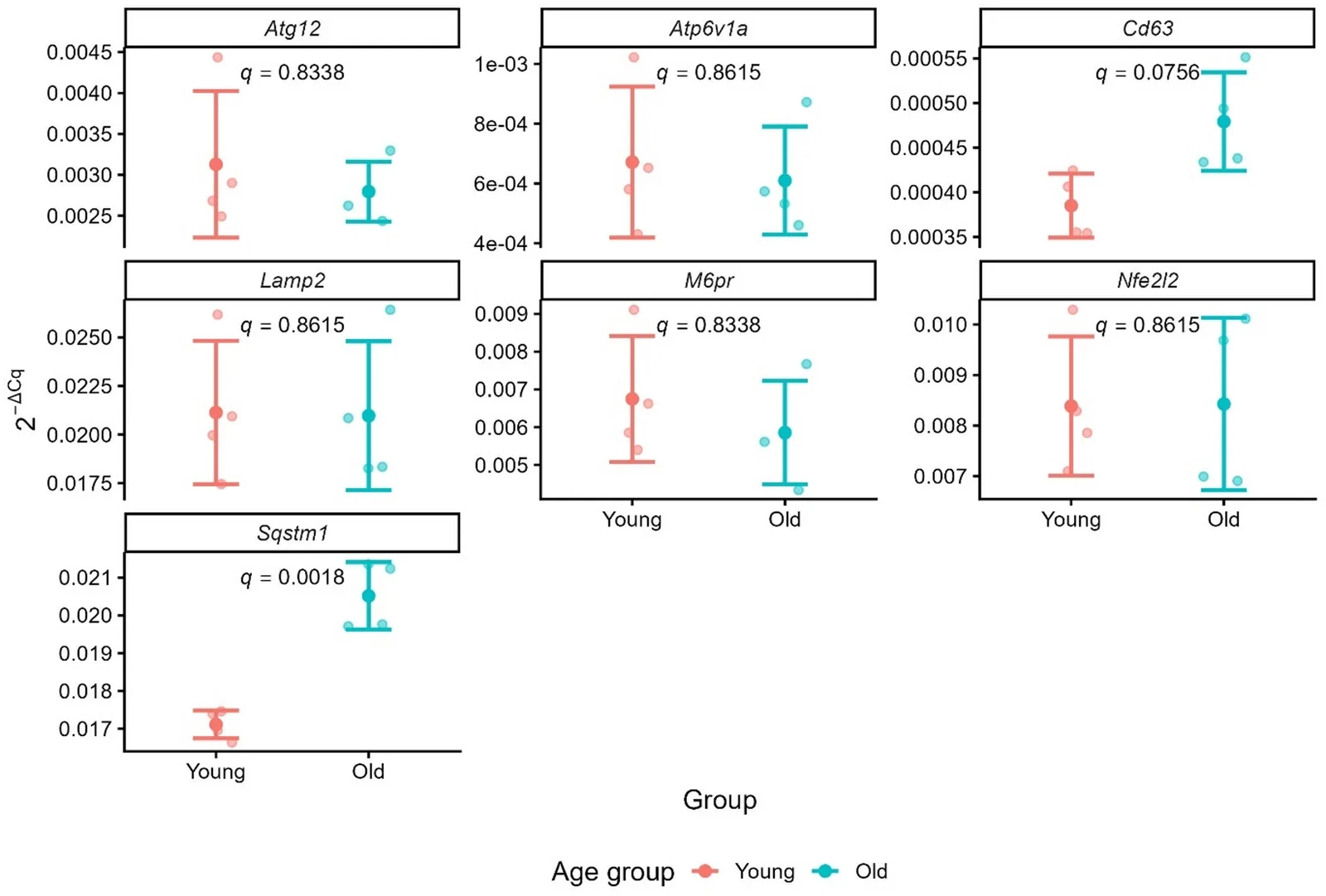
Gene expression in young and old mouse hearts (*n* = 4/group). Data represent transformed ΔCq values (2^−ΔCq^) calculated normalised to the housekeeping gene, *Gapdh*. Statistical comparisons performed using multiple unpaired *t-*tests with a two-stage Benjamini, Krieger and Yekutieli false discovery rate (FDR) correction. FDR-adjusted *p*-values (*q*-values) displayed on the graphs

Age-associated changes in the expression of lysosomal/autophagy-related genes were analysed in young and old mouse hearts by normalising target gene expression against that of the housekeeping gene, *Gapdh*, (ΔCq) in R (v4.4.1). As shown in Fig. 3, no significant differences (false discovery rate-adjusted *p* (*q*) > 0.5) in the expression of the lysosomal and autophagy genes *Atg12*, *Atp6v1a*, *Lamp2* and *M6pr*, or the antioxidant gene *Nfe2l2*, were identified. However, we noted increases in the expression of *Cd63* (*q* = 0.0756) and *Sqstm1* (*q* = 0.0018) in old hearts relative to young hearts. Full statistical outputs are provided in Supplementary Table S3.

## Discussion

This study investigated age-associated changes in cardiac lysosomal properties using a novel, whole-organ IVIS-based Lysotracker imaging approach, complemented by RT-qPCR analysis of selected lysosomal and autophagy-related genes. A key objective was to establish IVIS as a rapid, non-destructive method for quantifying acidic vesicle abundance in intact, *ex vivo* hearts prior to molecular interrogation. By integrating IVIS into this workflow, we aimed to determine whether this imaging modality could sensitively detect age-related shifts in acidic vesicle abundance before resolving underlying molecular changes. Our central hypothesis was that ageing would alter both the abundance of acidic organelles and the acidification machinery that supports them, leading to measurable differences in Lysotracker uptake and transcriptional profiles. Because Lysotracker accumulates in a protonated form within acidic compartments and cannot, on its own, resolve lysosomes from late endosomes, whole-organ signal is best interpreted as a proxy for the overall pool (number/volume) of acidic vesicles rather than absolute pH values [24]. Using an optimised dual-sided 2D imaging strategy (~ 5 s per orientation; ~10 s total per heart), we observed no significant age-related differences in global Lysotracker fluorescence, indicating that the overall pool of acidic organelles is preserved in this aged group (18 month old hearts). This interpretation is reinforced by the stable expression of *Atp6v1a*, a catalytic v-ATPase subunit required for proton translocation and lysosomal acidification [25], and *Lamp2*, a lysosomal membrane protein that contributes to structural stability and chaperone-mediated autophagy [5, 27]. Examining lysosome number in ageing hearts may be important to determine whether changes in quantity contribute to age-related decline, and the finding of stable numbers directs attention toward potential alterations in lysosomal function or efficiency instead.

The choice of fast 2D imaging was deliberate. While the IVIS Spectrum platform supports 3D fluorescence tomography [28], extended acquisition time risks *ex vivo* pH drift and probe signal decay. By imaging both anterior and posterior orientations, we maximised fluorescence capture while minimising artefacts, enabling confident assessment of whole-heart acidic-vesicle burden.

We also observed significantly higher Lysotracker signal in atrial regions compared to ventricles. This aligns with evidence that atrial cardiomyocytes are enriched in acidic vesicular stores, including atrial granules that interface with sarcoplasmic reticulum and mitochondria, supporting the atrium’s specialised role in Ca^2+^ handling and metabolic regulation [29]. Complementing this, our group previously [9] demonstrated that lysosomal Ca^2+^ release pathways strongly influence atrial electrophysiology, linking acidic organelle signalling to rhythm control. Together, these studies validate the sensitivity of IVIS Lysotracker imaging in capturing physiologically meaningful spatial differences in acidic organelle distribution.

Although no overall age effect was detected, we noted batch-to-batch variability in Lysotracker signals. In Batch 2, fluorescence was slightly lower. Biological differences may also have contributed, since young mice in Batch 2 were 4 months old compared with 2 months in Batches 1 and 3. Importantly, imaging conditions were kept constant across experiments, and batch was included as a fixed effect in the statistical model to account for this variability. These measures ensure that our conclusion, that the acidic-vesicle pool and acidification capacity are maintained with ageing, is robust and not confounded by procedural or biological variability. A limitation of IVIS fluorescence imaging is its depth-dependent signal attenuation due to tissue absorption and scattering. Although the murine left ventricular wall thickness (~ 1–1.5 mm) lies within the reported effective detection depth of IVIS (~ 2–5 mm) [30–32], photon propagation is strongly surface-weighted, resulting in preferential detection of fluorophores located in superficial myocardial layers. Consequently, Lysotracker signal likely reflects predominantly epicardial cardiomyocytes rather than uniformly sampling the full transmural myocardium. IVIS measurements should therefore be interpreted as semi-quantitative and depth-biased, providing relative comparisons rather than absolute estimates of total myocardial lysosomal content. Furthermore, changes in tissue composition or wall thickness associated with ageing may influence signal intensity independently of lysosomal biology. These considerations underscore that IVIS Lysotracker imaging is best suited for longitudinal or groupwise comparisons under similar imaging geometries, and complementary ex vivo or histological validation remains important for assessing transmural lysosomal distribution.

Our qPCR analysis revealed minimal transcriptional remodelling, in line with preserved whole-heart Lysotracker signal. However, as the assessment of autophagy-associated gene expression cannot measure post-translational modification and degradation of autophagy machinery, this approach may not fully capture the extent of cardiac autophagic function [33]. Therefore, minor upregulation of *Sqstm1* (p62), a selective-autophagy adaptor central to proteostasis and oxidative stress responses, and *Cd63*, a marker of late endosomes and MVBs, may be indicative of enhanced autophagy activity [34, 35]. *Cd63* upregulation may also reflect changes in vesicular trafficking and extracellular vesicle (EV) release, processes previously linked to ageing and intercellular signalling [36, 37]. Given the stable expression of *Lamp2* and *Atp6v1a*, these changes are likely to be consistent with increased autophagy demand rather than impaired degradation [5, 27, 38], although functional assays and assessment of autophagic protein expression are required to support this. Furthermore, as Lysotracker preferentially labels highly acidic lysosomes, and late endosomes are less acidic, the lack of fluorescence increase potentially suggests that late endosomes (measured via *Cd63*) contribute minimally to the whole-heart signal [24].

IVIS could be used as a high-throughput platform to monitor dynamic physiological or metabolic responses in isolated perfused hearts, building on its capacity to provide quick, whole-organ evaluation of fluorescent signals in intact hearts. To measure changes in autophagy, oxidative stress, Ca^2+^ handling, or mitochondrial activity under controlled Langendorff perfusion conditions, for instance, isolated hearts labelled with fluorescent or chemiluminescent metabolic probes could be imaged at multiple time points. This would allow for direct correlation between optical signals and ex vivo functional readouts and complement depth-limited whole-organ imaging. By using IVIS’s speed and sensitivity to connect molecular fluorescence signals with conventional physiological measures, this method would expand its usefulness beyond static ex vivo observations. Future studies should focus on three priorities. First, clarify organelle-specific contributions by combining IVIS imaging with ratiometric pH probes, compartment-specific labelling (e.g., LAMP2 vs. CD63), and V-ATPase inhibition assays to distinguish lysosomes from late endosomes [24]. Second, resolve cell-type and regional drivers of transcriptional changes using spatial transcriptomics and single-nucleus RNA-seq, particularly in atrial regions where Lysotracker signals were highest. This builds on findings that atrial cardiomyocytes contain specialised acidic vesicular stores that influence electrophysiology via lysosomal Ca^2+^ signalling [9, 29]. Finally, functional assays measuring autophagic flux, cathepsin activity, and extracellular vesicle release should determine whether subtle age-associated changes in *Sqstm1* expression reflects increased autophagic function or impaired clearance [27, 38]. Together, these approaches will define the mechanisms linking lysosomal Ca^2+^ signalling and atrial specialisation to cardiac ageing and age-related cardiac diseases such as AF, informing precision strategies to maintain lysosomal homeostasis.

The scope of physiological parameters measurable with IVIS extends beyond acidic vesicle abundance and depends on probe selection. These include intracellular pH, reactive oxygen species, mitochondrial membrane potential, metabolic activity, enzymatic activity, and aspects of calcium-associated signalling using fluorescent reporters. In cardiac applications, this enables integration of whole-organ imaging with functional readouts in ex vivo perfused systems. However, these measurements remain semi-quantitative and are constrained by probe specificity, optical attenuation, and limited spatial resolution.

### Limitations

This study has several limitations. First, IVIS fluorescence imaging is inherently depth-limited and surface-weighted. Because both excitation and emission light are strongly attenuated in cardiac tissue, the Lysotracker signal predominantly reflects the epicardial layer, and potential age-related alterations within deeper myocardial regions may have gone undetected.

Second, Lysotracker fluorescence reflects lysosomal acidity rather than degradative function or total lysosome number. Thus, changes in lysosomal pH or enzyme activity could occur without corresponding differences in fluorescence intensity, limiting interpretation of lysosomal function.

Third, bulk RT-qPCR analysis of *Lamp2* provides an averaged measure of gene expression across heterogeneous cardiac cell types. Consequently, distinct transcriptional alterations within specific cardiac cell populations, such as cardiomyocytes, fibroblasts, or endothelial cells, are not resolved. Additionally, because we did not separate atrial and ventricular tissue for analysis, any chamber-specific differences in lysosomal or autophagy-related gene expression may have been obscured.

Fourth, the use of *Lamp2* as a single lysosomal marker does not capture the full scope of lysosomal biogenesis or degradative capacity. Inclusion of additional lysosomal or autophagy-related genes (e.g., *Lamp1*,* Ctsd*,* Tfeb*) and protein or activity-based assays would yield a more comprehensive assessment.

Finally, all imaging and molecular analyses were conducted ex vivo, where altered perfusion, pH, and metabolic state may influence Lysotracker uptake and retention. Future studies integrating confocal or super-resolution imaging, immunostaining for multiple lysosomal markers, enzyme activity assays, and single-cell or spatial transcriptomics would help delineate cell-type specific and functional lysosomal remodelling during cardiac ageing.

## Conclusion

Taken together, these findings support a model in which the abundance of acidic vesicles and their acidification capacity are maintained with age. It is possible that lysosome-associated regulatory pathways may undergo adaptive remodelling but this needs further validation and exploration. Physiologically, these adaptations occur alongside atrial enrichment of acidic vesicular stores [29] and lysosomal Ca^2+^ signalling contributions to atrial excitability [9], highlighting the dynamic regulation of lysosome-associated pathways during cardiac ageing. However, we cannot say with certainty the true imaging depth achieved by IVIS lysotracker imaging in relation to its optical penetration, while bulk RT-qPCR averages expression across multiple cardiac cell types may not adequately reflect autophagic function. Consequently, subtle or cell-type–specific alterations in lysosomal function may have gone undetected. The close spatial proximity of lysosomes to the sarcoplasmic reticulum in myocytes [9, 13], highlights a potential structural and functional coupling that may influence intracellular Ca^2+^ dynamics. Investigating the quantity of acidic vesicles and their acidification capacity will provide critical insight into how lysosomal dysfunctions contribute to disease progression as well as in ageing. Future studies employing older mice (e.g. 24 months or older), higher-resolution imaging, LAMP2 immunostaining, and assays of lysosomal enzyme activity will be important to resolve regional and cellular differences.

## Methods

### IVIS Imaging

#### Ethical approval & animal details

All animal procedures were conducted in accordance with institutional and national ethical guidelines and were approved by [University of Liverpool, Animal Welfare and Ethical Review Body (AWERB)]. Mice were euthanized using rising concentrations of carbon dioxide (CO_2_), in accordance with Schedule 1 methods as defined by the UK Animals (Scientific Procedures) Act 1986 and the Home Office guidelines. Hearts from 14 male C57BL/6J mice (2–4-month-old or 18-month-old; *n* = 7 per age group) were included in the fluorescence imaging analysis. A total of 16 animals were used across three imaging batches (purchased from Janvier-Labs and Charles River Laboratories). In batch 1, one old mouse was treated with buffer only and served as a negative control (no dye treatment) to confirm dye loading and IVIS instrument performance; this sample was not included in the quantitative statistical analysis. In addition, one young mouse was excluded from the analysis because it was incubated longer than intended and showed substantially higher signal intensity. An overview of batch composition is shown in Table 1. Corresponding individual animal body and heart weights are provided in Supplementary Table S4.

**Table 1:** Batch Structure and Control. Details of batch dates, group sizes, and included samples for the IVIS experiment. Y = young; O = old

Batch	Date	Group Sizes	Notes
1	13 MAY 2025	3 old/3 young	One excluded. Final (Y1-Y2, O1-O2, Control)
2	7 JUL 2025	3 old/3 young	All included (Y3–Y5, O3–O5)
3	4 AUG 2025	2 old/2 young	All included (Y6–Y7, O6–O7)

### Reagent Preparation

**LysoTracker™ Red DND-99** (Thermo Fisher Scientific, Cat. No. L7528; 1 mM stock) was diluted by adding 0.5 µL to 5 mL of sterile, ice-cold Balanced Phosphate Solution (BPS) and protected from light.

**Tyrode’s solution** (composition in Table 2) was thawed from pre-prepared aliquots stored at − 20 °C and supplemented with 0.1 mL heparin per ~ 40 mL total volume immediately before use. This solution was used for retrograde perfusion during heart preparation.

**Phosphate Buffered Saline** (ice-cold, Gibco #10010-015) was used for control treatment in Batch 1.

**Table 2:** Composition of Tyrode’s Solution. Composition of Tyrode’s solution showing concentrations (mM) of each component, adjusted to pH 7.4 with NaOH

Component	Amount (mM)
KCl	5
NaCl	135
NaH₂PO₄	0.33
Na-pyruvate	5
HEPES	10
Mannitol	15
Glucose	5
CaCl₂	2
MgCl₂	1

### Phantom Studies

A preliminary phantom experiment was conducted to validate excitation/emission filter settings and optimize fluorescence detection before mouse imaging. LysoTracker™ Red DND-99 (Thermo Fisher Scientific, Cat. No. L7528; 1 mM stock) and LysoTracker™ Green (Thermo Fisher Scientific, Cat. No. 497269; 1 mM stock) were each diluted in 5 mL of sterile, ice-cold PBS and kept on ice, protected from light. Samples were transferred to a black-bottom 96-well plate to minimize background fluorescence, reduce light scattering, and prevent signal saturation. Several excitation/emission filter combinations were tested during the dry run, and based on these results and manufacturer specifications, the 570/620 nm filter set was selected for all subsequent experiments. This setting closely approximates the LysoTracker™ Red excitation/emission maxima (577/590 nm) and provided optimal signal quality confirmed using phantom dye controls.

### Heart Collection, Imaging, and Post-Processing

Mice were euthanized in pairs to standardize timing. Hearts were rapidly dissected, weighed, and placed in ice-cold Tyrode’s solution containing heparin. Blood was collected immediately into Microvette^®^ 500 CAT tubes (SARSTEDT, Cat. No. 20.1343), allowed to clot for 30 min at room temperature, and centrifuged at 3,000 rpm for 15 min at 20 °C. Serum supernatants were snap-frozen in liquid nitrogen and stored at − 80 °C for subsequent analyses.

**Fig. 4:**
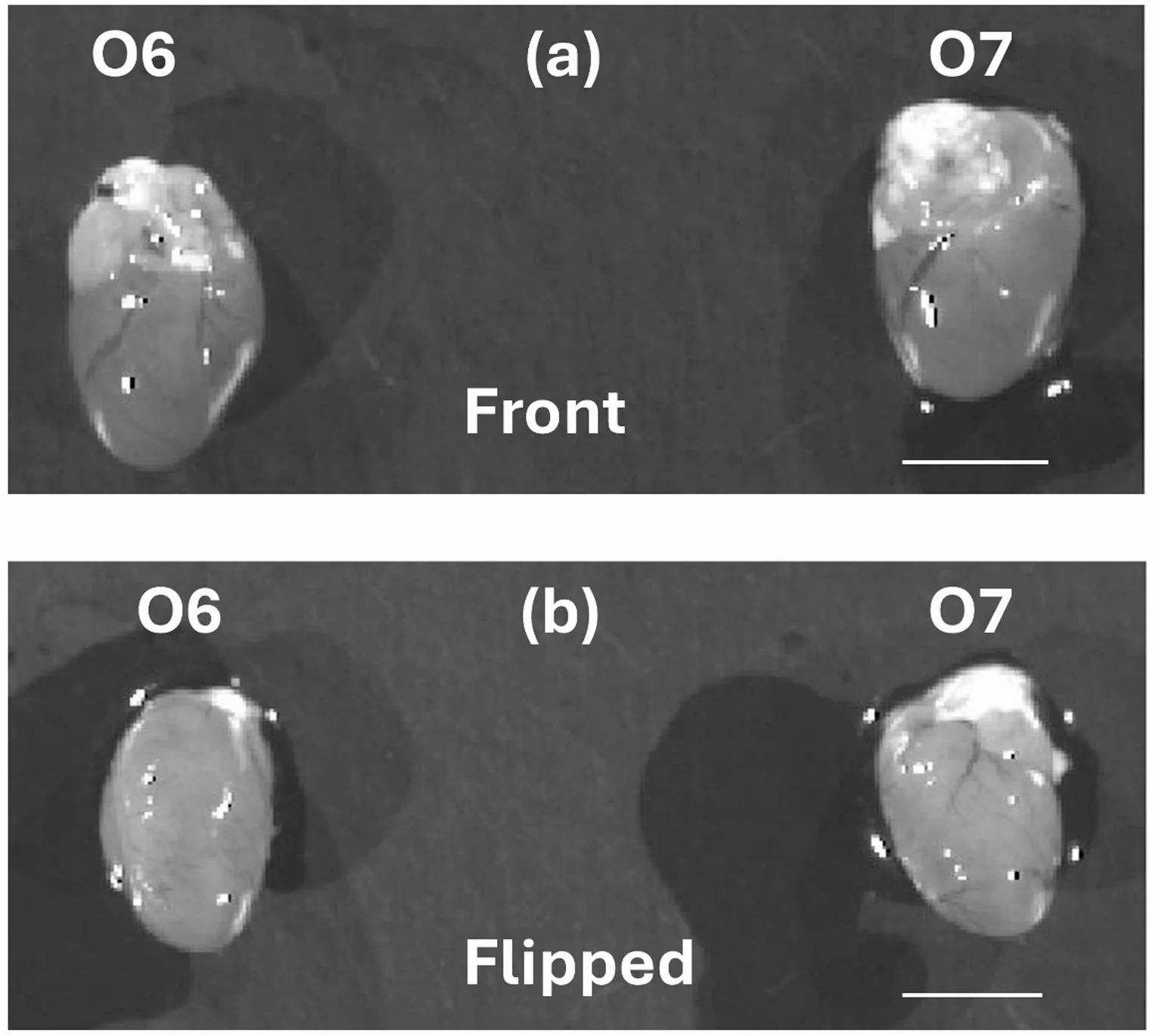
Orientation of mouse hearts during IVIS imaging. Representative image showing mouse hearts positioned in the front (anterior) and flipped (posterior) orientation within the IVIS^®^ Spectrum imaging chamber for fluorescence acquisition. O = old animal. Scale bar 0.5 cm

Epicardial fat on the heart surface was gently trimmed to expose the aorta, which was retrogradely perfused with Tyrode’s solution to remove residual blood. LysoTracker™ Red was then delivered via the aorta using a 1 mL syringe and plastic cannula, followed by 20 min incubation on ice, protected from light. Hearts were imaged on the IVIS^®^ Spectrum system (PerkinElmer) with the chamber maintained at 28–30 °C, using the 570/620 nm filter set. Each heart was imaged in both anterior (front) and posterior (flipped) orientations (Fig. 4).

After imaging, hearts were snap-frozen in liquid nitrogen and stored at − 80 °C for downstream qPCR analysis of selected lysosomal, late endosomal, and autophagy-related markers to complement IVIS measurements and evaluate age-related changes in lysosomal function and autophagy flux. Considering that LysoTracker™ Red accumulates in acidic intracellular compartments without distinguishing between lysosomes and late endosomes, signals were interpreted as a general proxy for acidic organelle abundance rather than a direct measure of lysosomal activity [24].

### Image Analysis

**Fig. 5:**
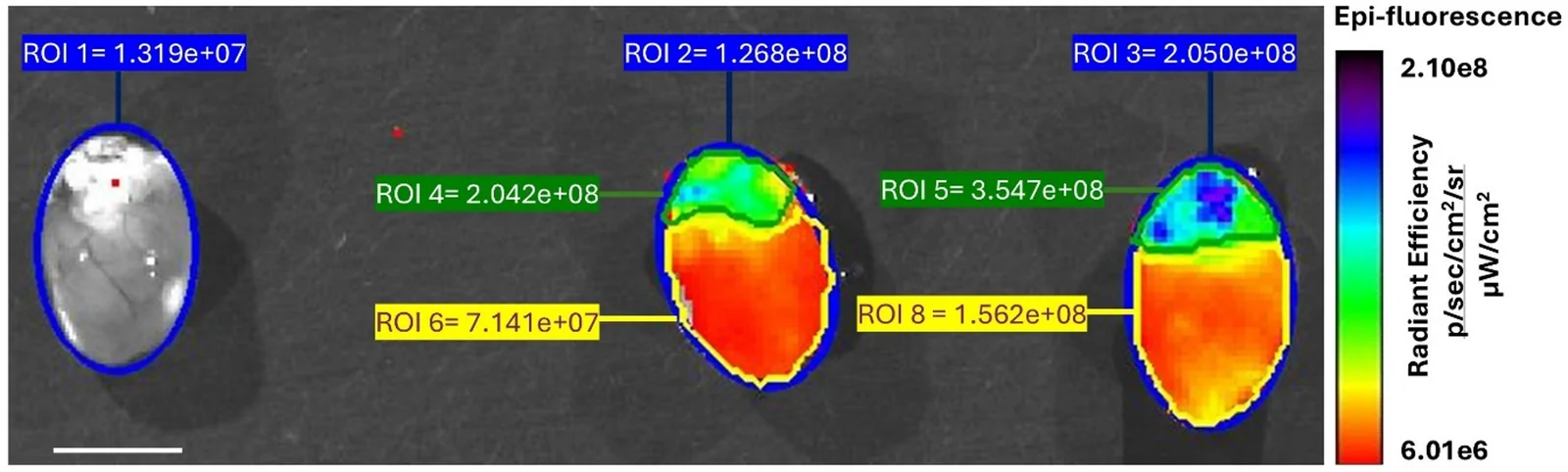
Representative IVIS^®^ Spectrum image from Batch 1 (Control, O1, O2; anterior view). Manually defined ROIs and corresponding fluorescence values. Blue = whole-heart, yellow = ventricle, green = atrium. Values represent Average Radiant Efficiency [p/s/cm²/sr]/[µW/cm²]. Scale bar 0.5 cm

Regions of interest (ROIs) were manually drawn using the Living Image^®^ v4.5.4 software, guided by anatomical landmarks with expert input. For each heart, ROIs for whole-heart, atrium, and ventricle were defined in both anterior and posterior orientations; paired orientation values were summed to yield one value per anatomical region per mouse. Fluorescence was quantified as Average Radiant Efficiency [p/s/cm²/sr]/[µW/cm²], enabling direct comparison across ROIs of different sizes. A representative example of ROI placement and signal distribution is shown in Fig. 5.

### Data Analysis

Fluorescence signals (Average Radiant Efficiency) were exported from Living Image^®^ (v4.7.3) and analyzed in R (v4.4.1). Anterior and posterior ROI signal intensity values for each region were summed to capture total signal (mitigating superficial bias inherent to IVIS imaging) and to minimize orientation-related variability. Linear mixed-effects models (lme4) included Age (young vs. old), Region (atrium vs. ventricle), and Batch were used as fixed effects, with Mouse ID as a random intercept. Both raw and log_10_-transformed data were tested; diagnostics supported the log_10_ model, which better satisfied assumptions. Estimated marginal means (EMMs) with 95% CIs were computed using the *emmeans* R package and back-transformed for interpretation. Final conclusions were based on the log_10_-transformed, batch-adjusted model.

### RT-qPCR Analysis

RNA was isolated from mouse hearts using the Monarch^®^ Spin RNA Isolation Kit (NEB, T2010) according to the manufacturer’s protocol and RNA concentration and purity were assessed using a NanoDrop™ ND-1000 spectrophotometer. Complementary DNA (cDNA) was synthesised from RNA using the LunaScript^®^ RT SuperMix Kit (NEB, E3010) and the following thermal cycling conditions: 2 min at 25 °C, 10 min at 55 °C, then 1 min at 95 °C. qPCR reactions were prepared using the Luna^®^ Universal qPCR Master Mix (NEB, M3003) and run on a QuantStudio ™ 7 Pro real-time PCR system in technical duplicates under the following thermal cycling conditions: 60 s at 95 °C, then 40 cycles of 15 s at 95 °C followed by 30 s at 60 °C. Following amplification, a melt curve was generated across a temperature range of 65–95 °C.

### Primer design and validation

Primers were designed for *Lamp2*, *Cd63*, *M6pR*, *Atp6v1a*,* Gapdh*, *Atg12*, *Nfe2l2*, and *Sqstm1* using NCBI Primer-BLAST, targeting exon–exon junctions to avoid genomic DNA amplification. Amplicon sizes (70–200 bp), GC content (40–60%), and melting temperatures (~ 60 °C) were optimized, while avoiding self-dimers, secondary structures, and primer–dimers (Supplementary Table S5). For this purpose, RNA was extracted from a young mouse sample (Y0, excluded from the imaging study) and a cDNA template standard curve ranging from 0 to 50 ng/µL was generated to validate primer efficiency. Acceptance criteria were defined as R^2^ ≥ 0.98, slope − 3.1 to − 3.6, and efficiency 90–110%. All primers met these thresholds except for *Atp6v1a* (R^2^ = 0.969) and *Cd63* *(R*^*2*^ *= 0.978*); however, these primers were accepted as these values were close to the defined threshold and the slope and efficiencies for these primers met the acceptance criteria (Supplementary Table S5).

### qPCR setup and controls

RNA and cDNA samples and qPCR reactions were prepared as previously described. RNA concentrations were standardised to 15 ng/µL. Non-template controls (NTC; water + master mix) and no-RT controls (NRT; RNA + water + no RT mix) were included in each run to exclude reagent contamination and genomic DNA carryover. All data were processed in R (v4.4.1). Target gene expression was normalised to that of the housekeeping gene, *Gapdh* and the effect of ageing on gene expression was assessed statistically in GraphPad Prism 10.2.3 using multiple unpaired *t-*tests with a two-stage Benjamini, Krieger and Yekutieli false discovery rate (FDR) correction.

All raw IVIS imaging data, qPCR measurements, and R analysis scripts supporting this study are openly available on Figshare at “https://doi.org/10.6084/m9.figshare.29984677 ”.

## Electronic Supplementary Material

Below is the link to the electronic supplementary material.

Supplementary Material 1

## Data Availability

Files have been uploaded onto figshare [https://doi.org/10.6084/m9.figshare.29984677]. The corresponding author can be contacted for any further details.
