# Supplementary material for: Age-related changes in lysosomal abundance in mouse hearts assessed by Lysotracker fluorescence imaging and autophagy gene expression analysis
Source: Int J Cardiovasc Imaging. 2026 May 19;42(9):1685–96. doi: 10.1007/s10554-026-03719-4 (PMC13582058; doi:10.1007/s10554-026-03719-4)
Supplement: Supplementary file 1 [file 10554_2026_3719_MOESM1_ESM.docx]

**SUPPLEMENTARY MATERIAL**

**Supplementary Figure S1.** Plots for the linear mixed-effects model assessing the effects of Age group, Region, and Batch on log10_{10}10-transformed IVIS signals.

**Top left:** Residuals vs. fitted values, showing no strong non-linear patterns but slight curvature, suggesting minor model misfit. **Top right:** Scale–location plot, indicating roughly constant variance across fitted values, with some spread differences at the extremes. **Bottom left:** Normal Q–Q plot of residuals, showing approximate normality with minor deviations in the tails. **Bottom right:** Normal Q–Q plot for random effects (Mouse_ID), indicating that random intercepts are approximately normally distributed. These diagnostics support the appropriateness of the model for the data, with no major violations of assumptions.

| **Region** | **Age_Group** | **Mean_log10** | **Lower95_log10** | **Upper95_log10** | **GeoMean** | **Lower95** | **Upper95** |
| --- | --- | --- | --- | --- | --- | --- | --- |
| Atrium | Young | 7.927 | 7.796 | 8.059 | 84500000 | 62500000 | 115000000 |
| Atrium | Old | 7.977 | 7.846 | 8.108 | 94800000 | 70100000 | 128000000 |
| Ventricle | Young | 7.506 | 7.375 | 7.637 | 32100000 | 23700000 | 43400000 |
| Ventricle | Old | 7.556 | 7.424 | 7.687 | 36000000 | 26500000 | 48600000 |

**Supplementary Table S1**. Estimated marginal means (EMMs) ± 95% confidence intervals for each Age × Region combination from the linear mixed-effects model, with both log10_{10}10-transformed and back-transformed (geometric mean) signal values. Back-transformed values are presented in photons/sec/cm²/sr for direct interpretability.

| **Effect** | **Sum Sq** | **Mean Sq** | **NumDF** | **DenDF** | **F value** | **Pr(>F)** |
| --- | --- | --- | --- | --- | --- | --- |
| **Age_Group** | 0.0023 | 0.0023 | 1 | 10 | 0.3767 | 0.5531 |
| **Region** | 1.2429 | 1.2429 | 1 | 13 | 207.3086 | 0 |
| **Batch** | 0.2075 | 0.1037 | 2 | 10 | 17.3036 | 0.0006 |

**Supplementary Table S2.** Full ANOVA results from the linear mixed-effects model testing the effects of Age group, Region, and Batch on log10_{10}10-transformed IVIS signals, with Mouse ID as a random intercept. Values include degrees of freedom (df), F-statistics, and *p*-values for each fixed effect.

|  | **P value** | **t ratio** | **DF** | **q value** |
| --- | --- | --- | --- | --- |
| **Atg12** | 0.5504 | 0.6325 | 6 | 0.8338 |
| **Atp6v1a** | 0.7484 | 0.3359 | 6 | 0.8615 |
| **Cd63** | 0.0250 | 2.970 | 6 | 0.0756 |
| **Lamp2** | 0.9470 | 0.06935 | 6 | 0.8615 |
| **M6pr** | 0.4225 | 0.8606 | 6 | 0.8338 |
| **Nfe2l2** | 0.9951 | 0.006422 | 6 | 0.8615 |
| **Sqstm1** | 0.0003 | 7.451 | 6 | 0.0018 |

**Supplementary Table S3.** Summary of multiple unpaired *t*-tests and Benjamini, Krieger and Yekutieli FDR correction testing the effects of age group ΔCq values measured by RT-qPCR. Values include unadjusted *p*-values, test statistics (t ratio), degrees of freedom (DF), FDR-adjusted *p*-values (*q*-value) for each gene.

| **Mouse ID** | **Animal Weight (g)** | **Heart Weight (g)** |
| --- | --- | --- |
| O1 | 29.4 | 0.20 |
| O2 | 33.6 | 0.27 |
| O3 | 36.78 | 0.36 |
| O4 | 36.04 | 0.26 |
| O5 | 36.59 | 0.26 |
| O6 | 31.7 | 0.25 |
| O7 | 33.6 | 0.30 |
| Y1 | 25.8 | 0.22 |
| Y2 | 21.8 | 0.28 |
| Y3 | 31.64 | 0.26 |
| Y4 | 30.56 | 0.39 |
| Y5 | 30.48 | 0.26 |
| Y6 | 20.19 | 0.18 |
| Y7 | 25.69 | 0.18 |
| Control | 32.8 | 0.33 |

**Supplementary Table S4**. Body and heart weights of all mice used in IVIS experiments. Animal weight and heart weight were recorded immediately after sacrifice for each mouse in the Old (O1–O7) and Young (Y1–Y7) groups, with one Control sample included. Values are presented in grams (g).

| **Gene** | **Forward primer (5′→3′)** | **Reverse primer (5′→3′)** | **Slope** | **R²** | **Efficiency (%)** |
| --- | --- | --- | --- | --- | --- |
| Gapdh | TGTCCGTGTGGATCTGAC | CCTGCTTCACCACCTTCTTG | –3.32 | 0.994 | 100.0 |
| Atg12 | TAAACTGGTGCCCTCGGAAC | ATCCCCATGCCTGGGAATTG | –3.28 | 0.998 | 102.0 |
| Nfe2l2 | CATGATGGACTTGGAAGTGC | CCTCCAAAGGATGTCAATCAA | –3.47 | 0.999 | 94.0 |
| Sqstm1 | CTCAGCCCTCTAGGCATTG | AGCTGCTTGCGTGAGTGTTTA | –3.30 | 0.998 | 101.0 |
| Atp6v1a | GTGAGGTGCGACAGGTAAA | AATTTCTCCAACCAGCTCGC | –3.43 | 0.969 | 95.7 |
| Lamp2 | CCTGCCACAACCAACTTCAC | TGGAAATGTTGAAAGCTGAGCC | –3.57 | 0.987 | 90.6 |
| Cd63 | CACACGGGAGAAAGGCCCAA | ATCAATCCCACTGCACAGGC | –3.20 | 0.978 | 105.4 |
| M6pr | CCTGTTCACCAGAGGTCTCAC | CACAAAGTCACAACCATCAGCTA | –3.57 | 0.989 | 90.5 |

**Supplementary Table S5.** Validated primer sequences and performance parameters used for qPCR. All primers demonstrated acceptable qPCR performance, with slopes between –3.1 and –3.6 and efficiencies between 90–110 %. R² values were ≥0.98 for all assays, with the exception of *Atp6v1a* (R^2^ = 0.969) and *Cd63* (R² = 0.978), which fell just below the R^2^ cut off but retained excellent efficiency, supporting their suitability for use.
